# The Coexpression of Reelin and Neuronal Nitric Oxide Synthase in a Subpopulation of Dentate Gyrus Neurons Is Downregulated in Heterozygous Reeler Mice

**DOI:** 10.1155/2010/130429

**Published:** 2010-09-01

**Authors:** Raquel Romay-Tallón, Iria G. Dopeso-Reyes, April L. Lussier, Lisa E. Kalynchuk, Hector J. Caruncho

**Affiliations:** ^1^Biofarma Research Group, Department of Cell Biology, Faculty of Biology, University of Santiago de Compostela, Santiago de Compostela, 15782 Galicia, Spain; ^2^Neural Systems and Plasticity Research Group, Department of Psychology, University of Saskatchewan, Saskatoon, SK, Canada S7N 5E5

## Abstract

Reelin is an extracellular matrix protein expressed in several interneuron subtypes in the hippocampus and dentate gyrus. Neuronal nitric oxide synthase (nNOS) is also expressed by interneurons in these areas. We investigated whether reelin and nNOS are co-localized in the same population of hippocampal interneurons, and whether this colocalization is altered in the heterozygous reeler mouse. We found colocalization of nNOS in reelin-positive cells in the CA1 stratum radiatum and lacunosum moleculare, the CA3 stratum radiatum, and the dentate gyrus subgranular zone, molecular layer, and hilus. In heterozygous reeler mice, the colocalization of nNOS in reelin-positive cells was significantly decreased only in the subgranular zone and molecular layer. The coexpression of reelin and nNOS in several hippocampal regions suggests that reelin and nNOS may work synergistically to promote glutamatergic function, and the loss of this coexpression in heterozygous reeler mice may underlie some of the behavioral deficits observed in these animals.

## 1. Introduction

Reelin is a large extracellular matrix protein that plays an important role in regulating neural migration and synaptogenesis during development. It is also a key component of synaptic plasticity in the adult brain (see [1, 2], as recent reviews). As such, reelin promotes dendritic development [3–5] and synaptogenesis [6, 7], contributes to the maturation of dendritic spines [8, 9], increases NMDA receptor subunit activity [10, 11], and enhances long-term potentiation [12–14]. Reelin influences neural plasticity primarily through activation of VLDLR and ApoER2 receptors (for a review, see [2]), but it also increases translation of selective mRNAs in dendritic spines by binding to integrins located in the plasma membrane. One example of this is the recent observation that reelin can increase the translation of activity-related cytoskeletal protein (Arc) thereby influencing spine maturation and stability [9]. In addition, reelin also induces the clustering of its receptor [15], and increases the number of intramembrane particles (i.e., transmembrane proteins) in synaptosomal membranes [16].

One purpose of the present study was to determine whether nitric oxide is expressed in reelin-positive cells located in the hippocampus and dentate gyrus. Nitric oxide (NO) is a gaseous messenger that plays an important regulatory role in many of the same forms of hippocampal plasticity as those described above for reelin. For example, NO regulates NMDA receptor activity, enhances long-term potentiation, and increases the formation of dendritic spines in the adult brain (see [17]). NO expression in neurons arises from the activity of neuronal nitric oxide synthase (nNOS). These nNOS-positive neurons can be localized via immunohistochemical detection of nicotinamide adenine dinucleotide phosphate diaphorase (NADPHd), which acts as a coenzyme for nNOS (reviewed in [17]). Given that both reelin and nNOS have been implicated in NMDA receptor regulation and long-term potentiation within hippocampal circuits, we hypothesized that nNOS may be expressed by reelin-positive interneurons in specific hippocampal regions, thus facilitating hippocampal plasticity. 

A second purpose of this work was to determine whether the loss of reelin signalling also impacts nNOS expression in the same cells. Deficits in reelin levels and a loss of reelin-positive cells are apparent in brain pathologies such as schizophrenia, depression, and epilepsy and this may be accompanied by alterations in the nitrinergic system. For example, examination of postmortem tissue from patients with schizophrenia revealed a 50% decrease in reelin levels [18–23], a decrease dendritic spine density [24–30], and a decrease in the number of NADPHd- or NOS-positive neurons [31–35]. Importantly, reelin haploinsufficient heterozygous reeler mice (HRM), which express about 50% of normal brain levels of reelin, also show deficits in cortical dendritic spines and GAD67 expression, a decrease of NADPHd-positive neurons in the cortical white matter [36–39], and profound disturbances in hippocampal synaptic plasticity and long-term potentiation [12, 40, 41]. In addition to schizophrenia, reelin deficiencies have been observed in a stress-based animal model of depression [42] and the activity of the nitrinergic system appears to be important for the development of stress and depression symptoms [43]. Finally, a deficiency in hippocampal reelin expression may also be involved in the dysregulation of hippocampal neurobiology underlying the formation of seizures [44–46]. NO and nNOS have been implicated in the initiation of hippocampal seizures [47]. 

The potential synergistic action of reelin and nNOS in the adult hippocampus has not been studied, but in the olfactory bulb, nNOS-positive neurons express the reelin receptor ApoER2 and target some reelin-positive cells. Surprisingly, there is no neuronal colocalization of both reelin and nNOS in this region in adults [48]. However, during brain development, Cajal-Retzius cells that characteristically express reelin also express nNOS in both rodents [49, 50] and humans [51]. In addition, nitric oxide is expressed by some pyramidal-basket neurons in the dentate subgranular cell layer [52], and we have previously shown that a single layer of pyramidal-basket cells in this region also express reelin [53].

Based on the overall pattern of findings discussed here, we hypothesized that there may be a functional connection between reelin and nNOS in regulating dendritic spine plasticity in hippocampal brain regions, and that this connection may be dysregulated under pathological conditions that affect the hippocampus. To begin to study this hypothesis, we examined the colocalization of reelin and nNOS throughout the hippocampus and dentate gyrus, where both reelin and nNOS appear to be expressed by selective subtypes of GABAergic interneurons [52–57]. We also assessed whether the colocalization of reelin and nNOS-positive neurons under normal circumstances is altered in the heterozygous reeler mouse.

## 2. Materials and Methods

### 2.1. Animals and Tissue Processing

We used 16 adult male mice in this experiment: 8 wild type mice (WTM) and 8 heterozygous reeler mice (HRM) (B6C3Fe-a/a-Reln^rl^). The animals were obtained from heterozygous reeler pairs (Jackson Laboratory, Bar Harbor, ME) maintained in our colony at the University of Santiago de Compostela, Spain. Genotyping was performed using the polymerase chain reaction (PCR) technique as described previously [58]. The following primers were used: 5′-TAA TCT GTC CTC ACT CTG CC-3′, 3′-ACA GTT GAC ATA CCT TAA TC-5′, 3′-TGC ATT AAT GTG CAG TGT TGT 5′. The PCR products were analyzed in a 2% agarose gel. The product from wild type mice DNA is 266 bp long and the product from heterozygous reeler mice is 363 bp long.

For histological studies, the animals were anesthetized with 15% chloral hydrate, followed by transcardial perfusion with saline and 4% paraformaldehyde in 0.1 M phosphate buffer (PB) at room temperature. The brains were removed from the cranium and postfixed for 24 hours at 4°C in the same fixative. Subsequently, the brains were rinsed with 0.1 M PB and cryoprotected with 30% sucrose in 0.1 M PB. The brains were sectioned at 35 *μ*m in a cryostat at −20°C, and the sections were stored in antifreeze solution containing ethylene glycol/glycerol in 0.1 M PB, until immunolabelling.

### 2.2. Immunohistochemistry

Immunohistochemistry was performed on free-floating coronal sections corresponding to the coordinates −1.46 mm to −2.06 mm from bregma [59]. The sections were rinsed several times in Tris buffered saline (pH 7.4, TBS), followed by an overnight incubation at room temperature with mouse monoclonal antireelin (1 : 150, Chemicon, #MAB5364) and rabbit polyclonal anti-nNOS (1 : 250, Sigma, #N7280) antibodies diluted in a solution containing 15% normal goat serum (NGS), 0.5% Triton X-100, and 1% BSA diluted in TBS. Thereafter, the sections were rinsed with TBS and incubated for 2 hours at room temperature with fluorescent-labelled secondary antibodies: Alexa 488 goat antimouse and Alexa 546 goat anti-abbit. The secondary antibodies were diluted in a solution containing 15% NGS, 0.5% Triton X-100, and 1% BSA in TBS. To assess nonspecific background labelling, we carried out control experiments with the omission of primary antibodies from the incubation cocktail and this resulted in a total absence of labelling.

### 2.3. Data Analyses and Statistics

Confocal images were obtained using a spectral confocal microscope (Leica TCS SP2) in 1 *μ*m thick virtual slices. The analysis of double-labelled neurons in virtual slices of 1 *μ*m thickness allowed us to identify double-labelled cells more accurately compared to the more standard practice of counting cells in whole sections of 35 *μ*m thickness. This decreased the probability of counting artifacts due to false positives. 

Colocalization of reelin-positive and nNOS-positive cells was assessed in the following layers in the hippocampus: stratum oriens (SOs), stratum radiatum (SR), stratum lacunosum moleculare (SLM), and pyramidal cell layer (SP) within the CA1 and CA3 subfields; as well as in the dentate gyrus molecular layer (ML), subgranular zone (SGZ), and the hilus. Within the confocal images, we counted cells that expressed both reelin and nNOS. For each animal, we counted cells in three sections per brain and three random cell fields per layer, using a 40X objective lens. Data are expressed as the mean ± SEM of the percentage of reelin-positive cells that coexpress nNOS. 

For the statistical analyses, we used SPSS software, version 11 (Chicago, IL, USA). Group differences in the percentage of cells showing reelin and nNOS colocalization were examined using separate two-tailed t-tests for each region of the hippocampus. The criterion for statistical significance was set at *P* < .05.

## 3. Results

**Figure 1:**
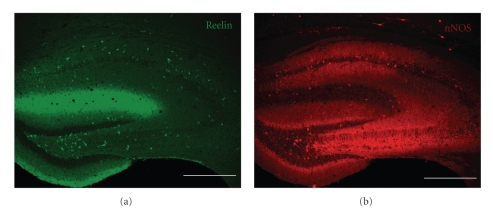
Coronal section of the anterior hippocampus immunostained for reelin (a) and nNOS (b). Note that both markers are primarily expressed by some interneuron subtypes in different hippocampal layers, but not by pyramidal neurons or granule cells. Calibration bars: 300 *μ*m.

Figure 1 shows the overall pattern of reelin (Figure 1(a)) and nNOS (Figure 1(b)) immunoreactivity in the hippocampus of wildtype mice. Reelin immunoreactivity (reln-ir) was located across a subset of nonpyramidal neurons in the hippocampus and dentate gyrus. Reln-ir was primarily seen as cytoplasmic labelling limited to cell bodies and proximal processes. In addition, there was diffuse labelling in the hippocampus stratum lacunosum-moleculare, which probably represents reelin within the neuropil and extracellular space. nNOS immunoreactivity (nNOS-ir) was also observed in scattered nonpyramidal neurons in the hippocampus and dentate gyrus. This labelling was also cytoplasmic and somewhat weaker than what is typically seen in nNOS-ir cortical neurons (see Figure 1(b)).

**Figure 2:**
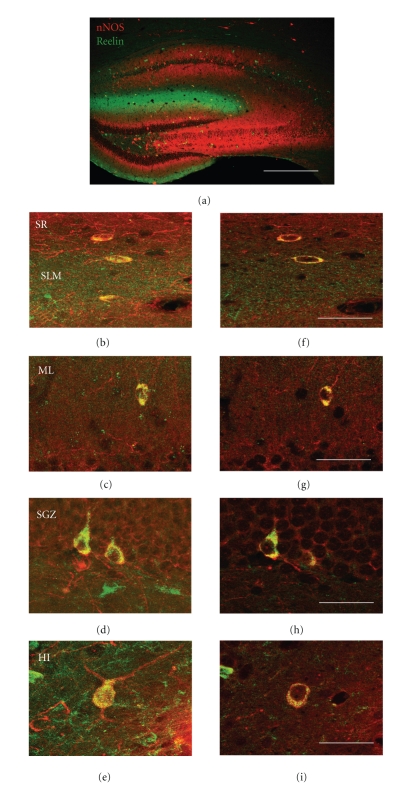
Overlay micrograph of double-immunostaining experiments showing colocalization of reelin (green) and nNOS (red) in the anterior hippocampus (a). High magnification micrographs of several hippocampal interneurons coexpressing reelin and nNOS (b–i). The left-side panels show the neurons as seen in 35 *μ*m sections whereas the right-side panels show a virtual section 1mm in thickness of these same neurons. The virtual slices allowed us to confirm the colocalization of both markers in the cytoplasm of the same neurons. SR (stratum radiatum), SLM (stratum lacunosum-moleculare), ML (dentate molecular layer), SGZ (dentate subgranular zone), and HI (hilus). Calibration bars: 300 *μ*m (a), 50 *μ*m (b–i).

Figure 2(a) shows an overlay of optical confocal images of both reln-ir and nNOS-ir across the whole hippocampus and several hippocampal subregions in the wildtype mice. The percentage of cells that expressed both reln-ir and nNOS-ir varied across the different hippocampal regions and layers that were quantified. High levels of colocalization between reln-ir and nNOS-ir were seen in the dentate gyrus. Note that the granule cell layer has no reelin-positive cells, so there was no possibility of co-localized expression of reelin and nNOS in this area. However, in the subgranular zone, about 25% of reln-ir cells expressed nNOS-ir (24.19 ± 2.92); in the hilus, about 20% of reln-ir cells expressed nNOS-ir (17.39 ± 3.90%); in the molecular layer, about 50% of reln-ir cells expressed nNOS-ir (49.28 ± 2.38). These data are shown in Figure 5. 

Within the hippocampus proper, we observed about 20% colocalization of reln-ir and nNOS-ir in the CA1 stratum radiatum (19.35 ± 2.43) and CA1 stratum lacunosum-moleculare (20.44 ± 1.72), and about 10% colocalization of reln-ir and nNOS-ir in the CA3 stratum radiatum (12.58 ± 1.96). There was no colocalization of reln-ir and nNOS-ir in the CA1 and CA3 pyramidal cell layers or stratum oriens. These data are also shown in Figure 5. 

Close examination of the images in Figure 2 revealed that the colocalization of reln-ir and nNOS-ir in the CA1 and CA3 stratum radiatum occurs primarily in small multipolar neurons (Figures 2(b) and 2(f)). Colocalization of both markers was also found in some round or fusiform small neurons in the CA1 stratum lacunosum-moleculare and the dentate gyrus molecular layer (Figures 2(c) and 2(g)), in a subpopulation of pyramidal-basket cells in the dentate gyrus subgranular zone (Figures 2(d) and 2(h)), and in some multipolar neurons in the hilus (Figures 2(e) and Figures 2(i)).

**Figure 3:**
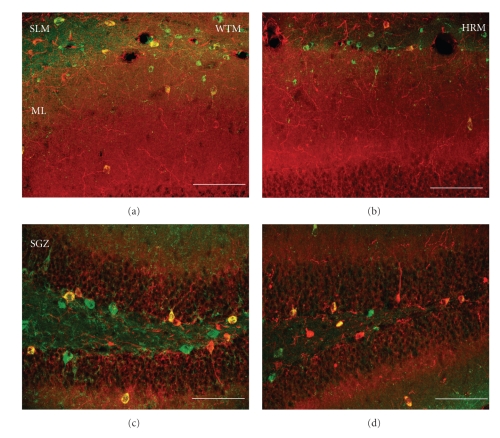
Representative micrographs of double immunolabelling of reelin (green) and nNOS (red) in wild type mice (WTM) and heterozygous reeler mice (HRM) in the CA1 stratum lacunosum-moleculare and dentate molecular layer (a, b), and the dentate subgranular zone and hilus (c, d). Note the decrease in the number of neurons coexpressing both markers in the heterozygous reeler mice dentate molecular layer and subgranular zone. Calibration bars: 75 *μ*m.

**Figure 4:**
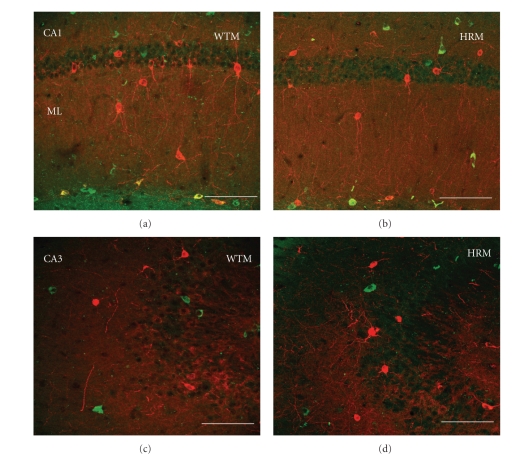
Representative micrographs of double immunolabelling of reelin (green) and nNOS (red) in wild type mice (WTM) and heterozygous reeler mice (HRM) in the CA1 (a, b) and CA3 hippocampal regions (c, d). Calibration bars: 75 *μ*m.

**Figure 5:**
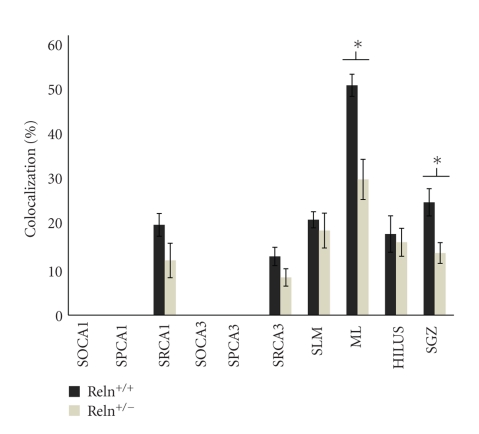
Histogram showing the percentage of reelin-ir cells that also show nNOS-ir. Note the decrease in the level of colocalization observed in the dentate molecular layer and subgranular zone of the heterozygous reeler mouse. SO (stratum oriens), SP (pyramidal cell layer), SR (stratum radiatum), SLM (stratum lacunosum-moleculare), ML (dentate molecular layer), and SGZ (dentate subgranular zone). Data are expressed as the mean ± SEM.

Once we established the existence of some colocalization of reln-ir and nNOS-ir in wildtype mice, we proceeded to study whether the pattern and extent of colocalization is altered in the heterozygous reeler mouse. Figures 3 and 4 show representative examples of reln-ir and nNOS-ir colocalization throughout the dentate gyrus and hippocampus whereas Figure 5 shows the quantitative data of this colocalization. The percentage of reln-ir cells that coexpress nNOS-ir was not significantly altered in any quantified regions of the hippocampus and dentate gyrus except for the molecular layer and subgranular zone (all *P*'s < .11). In the molecular layer and subgranular zone, the heterozygous reeler mice had significantly less colocalization of reln-ir and nNOS-ir (ML: *t*(13) = 3.93, *P* < .001; SGZ: *t*(13) = 3.01, *P* < .01). In practical terms, these differences amounted to about a 40% decrease in the percentage of reln-ir cells that coexpress nNOS in the molecular layer of heterozygous mice compared to wildtype mice and about a 45% decrease in the percentage of cells that coexpress nNOS in the subgranular zone of heterozygous mice compared to wildtype mice. 

## 4. Discussion

The results of this study provide the first data showing that a subset of reelin-positive cells throughout the hippocampus and dentate gyrus also express nNOS. They also revealed that this colocalization of reelin and nNOS is substantially decreased in heterozygous reeler mice. Reelin haploinsufficient heterozygous mice generally have reelin levels that are about 50% lower than those observed in wildtype mice [36]. However, despite the lower levels of reelin in these mice, reln-ir cells can still be detected with immunohistochemistry, albeit with a less intense signal (see Figures 3 and 4). This dictated our approach for measuring the potential colocalization of reelin and nNOS. We opted to assess the percentage of reln-ir cells that coexpress nNOS, rather than the percentage of nNOS-ir cells that coexpress reelin, because the former method eliminates the possibility of counting artifacts due to very low levels of reelin immunoreactivity. For example, it is possible that nNOS-positive cells might be considered reelin negative in the heterozygous mice due to very low levels of reelin staining, leading to false negatives. 

To our knowledge, there have been no previous studies of reelin and nNOS colocalization in the rodent hippocampus. Previous studies have focused on the olfactory bulb, showing that NOS-positive neurons in this region express receptors for reelin but no reelin itself [48]. Similarly, we have also failed to identify neurons showing both reln-ir and nNOS-ir in the cerebral cortex (unpublished data). However, the labelling of nNOS-ir in some somatostatin-ir pyramidal-basket cells in the dentate gyrus subgranular layer [52], together with our results indicating that a subset of these cells is reelin-positive [53], prompted us to speculate that perhaps the nNOS-ir and reln-ir cells were part of the same neuronal subpopulation. In addition, different interneuron GABAergic subtypes in different areas of the rodent hippocampus have been shown to express nNOS [60–63]. Our results demonstrate that we were partially right, in that about 25% of pyramidal-basket reln-ir cells in the dentate subgranular layer also show nNOS-ir. Interestingly, the colocalization of both markers is not restricted to this cell subpopulation, because we also saw nNOS-ir in about 20% of reln-ir cells in the stratum radiatum, stratum lacunosum-moleculare, and the hilus, and in about 50% of reln-ir cells in the dentate distal molecular layer. No colocalization of reelin and nNOS was apparent in the stratum oriens or pyramidal and granule cell layers (although in these last two areas, reln-ir cells are basically nonexistent). In the adult cortex and hippocampus, reelin is primarily observed on the surface of dendritic spines [8] after being secreted primarily by GABAergic interneuron subtypes impinging onto apical dendrites of pyramidal neurons [55]. Reelin also affects NMDA receptor activity [10, 11] and enhances hippocampal long-term potentiation [12–14]. All these actions are also promoted by nitric oxide (reviewed in [17]). The lack of colocalization of reln-ir and nNOS-ir in neurons within the stratum oriens that primarily contact basal dendrites of pyramidal neurons, and the partial colocalization of both markers in interneurons of the stratum radiatum, stratum lacunosum-moleculare, and dentate molecular layer, suggest that there could be a synergic action of both reelin and NO in strengthening some glutamatergic synapses onto the distal apical dendrite of pyramidal (or dentate granule cell layer) neurons, potentially facilitating long-term potentiation. Clearly, additional studies will be needed to evaluate this hypothesis.

A second issue is that of the colocalization of reelin and nNOS in a subpopulation of reln-ir cells in the subgranular zone. These cells are pyramidal-basket cells that impinge onto the cell body of granule neurons. It is possible that the release of reelin and NO in the subgranular zone, or by cells in the hilus, could influence the migration and maturation of newly born granule cells. Lussier et al. [42] recently showed that repeated exposure to high levels of glucocorticoids, which increases depression-like behavior and decreases hippocampal neurogenesis [64–66], significantly decreases the number of reelin-positive cells in the subgranular zone of the dentate gyrus [42]. Similarly, Pujadas et al. [67] reported that reelin overexpression increased hippocampal neurogenesis, increased synaptic contacts, and produced dendritic hypertrophy. These results provide some momentum for the idea that reelin-positive cells in the subgranular zone influence neurogenesis, but whether these reelin-positive cells are the ones that also contain nNOS and how exactly reelin and nNOS interact awaits further study. Preliminary studies from our laboratory have shown that heterozygous reeler mice show alterations in the maturation of newborn dentate granule cells, which could relate to possible disruptions in plasticity within the dentate gyrus. We have also found that heterozygous reeler mice are more vulnerable to the deleterious effects of corticosterone on reelin-positive pyramidal-basket cells and depressive-like behavior (Lussier et al., personal communication).

A final issue relates to the functional implications of a decrease in reelin and nNOS colocalization in heterozygous reeler mice. A previous study by Hermann et al. [68] showed a decline in nNOS protein, but not nNOS mRNA, in the olfactory bulb of reeler mice. Here, we report a decrease of about 40%–45% of reelin-positive cells that coexpress nNOS specifically in the dentate molecular layer and subgranular zone, but not in other hippocampal areas. Although this is not a functional study, it is tempting to propose that the specific alteration of reelin and nNOS colocalization in the dentate gyrus of heterozygous reeler mice could underlie some of the disturbances in synaptic plasticity that have been observed in these mice [12, 40, 41]. For example, Qiu et al. [40] found that heterozygous reeler mice are impaired on a contextual fear-conditioning task and also display deficits in long-term depression and long-term potentiation. The converse relationship is also true. Pujadas et al. [67] revealed that reelin overexpression provoked substantial increases in long-term potentiation. Importantly, nNOS knockout mice have a severe deficit in contextual fear conditioning [69, 70] and nNOS is required for the induction of long-term potentiation [71]. However, one should consider that most of these studies focus primarily on hippocampal CA1 long-term potentiation whereas our observations show a deficient reelin-nNOS colocalization in the dentate gyrus of the heterozygous reeler mouse. Therefore, it would be interesting to study potential alterations in long-term potentiation in the dentate gyrus of these animals, mostly taking into account that dentate gyrus long-term potentiation enhances adult dentate neurogenesis and the rate of new neuron survival and maturation [72–74], and that the induction of long-term depression suppresses this effect [75]. In any case, the fact that reelin and nNOS seem to be involved in similar types of hippocampal-dependent behavior and synaptic plasticity, combined with our observations here of colocalization of reelin and nNOS throughout the hippocampus and dentate gyrus and loss of colocalization when reelin is deficient, suggest that reelin and nNOS may work together to regulate some aspects of hippocampal function. 

In conclusion, the results of this study demonstrate that a subpopulation of hippocampal and dentate gyrus reelin-positive cells also express nNOS, and that this coexpression is decreased selectively in the dentate gyrus of heterozygous reeler mice. We propose that some of these alterations could be operative in the disturbed synaptic plasticity associated with a loss of reelin.
